# Building biologically relevant *in vitro* models of the human gut microbiota: methodological considerations, current challenges, and future perspectives

**DOI:** 10.3389/fmicb.2026.1949973

**Published:** 2026-10-15

**Authors:** Simon De Jaegher, Christine Podrini, Maria D’Aguanno, Francesco Santoro, Manuele Biazzo

**Affiliations:** 1 Laboratory of Molecular Microbiology and Biotechnology (LAMMB), Department of Medical Biotechnologies, University of Siena, Siena, Italy; 2 The BioArte Ltd., Life Science Park, San Gwann, Malta

**Keywords:** continuous bioreactor systems, gut microbial ecology, human gut microbiota, *in vitro* fermentation models, microbiome methodology, personalized microbiome interventions

## Abstract

The human gut microbiota (HGM) plays a pivotal role in human health by regulating metabolic, immune, and neurological processes and contributes to the susceptibility to various diseases. The increasing interest in microbiota-targeted therapies has highlighted the need for *in vitro* models capable of accurately reproducing the composition, function, and ecological dynamics of the intestinal microbial ecosystem. This review examines the methodological factors that determine the biological relevance of HGM *in vitro* models, emphasizing that reliable experimental outcomes depend not only on the choice of fermentation system (including batch and continuous fermentation systems), but also on the optimization of inoculum preparation, culture media, environmental control, and strategies that preserve microbial stability and reproducibility. We further discuss how these methodological decisions influence the interpretation of experimental results and determine the suitability of each model for different biological applications. The application of these models in dietary, prebiotic, probiotic, and pharmacological interventions is explored, demonstrating their potential to predict microbiota responses and elucidate mechanistic links between microbial activity and host responses. We propose that rigorous methodological standardization, rather than increasing technological complexity alone, represents the principal requirement for generating biologically relevant and reproducible *in vitro* gut microbiota models. Finally, we discuss emerging host-microbe co-culture systems, along with the integration of multi-omics and computational approaches, which are expected to expand the physiological relevance and translational applicability of these models. Unlike reviews primarily organized around individual fermentation technologies, this review adopts a workflow-based perspective and examines how methodological decisions interact to determine ecological fidelity, functional stability and reproducibility.

## 1 Introduction

The human body contains several complex ecosystems consisting of bacteria, archaea, viruses, protozoa and fungi (Matijašić et al., 2020). Collectively referred to as the microbiota, these communities are primarily located in the gastrointestinal and respiratory tracts, on the skin, and within the vaginal environment. The gastrointestinal (GI) tract, particularly the colon, harbors the largest and most diverse microbial community in the human body (D’Angelo et al., 2017). These microorganisms interact with each other, as well as with their host in a symbiotic way (Kamada et al., 2013). Despite marked interpersonal variability, the healthy human gut microbiota is generally dominated by anaerobic taxa belonging primarily to four major phyla: Bacillota, Bacteroidota, Actinomycetota, and Proteobacteriota (Tap et al., 2009; The Human Microbiome Project Consortium, 2012). The relative abundance of the dominant gut microbiota phyla and representative associated species in Western populations is illustrated in Figure 1.

**Figure 1:**
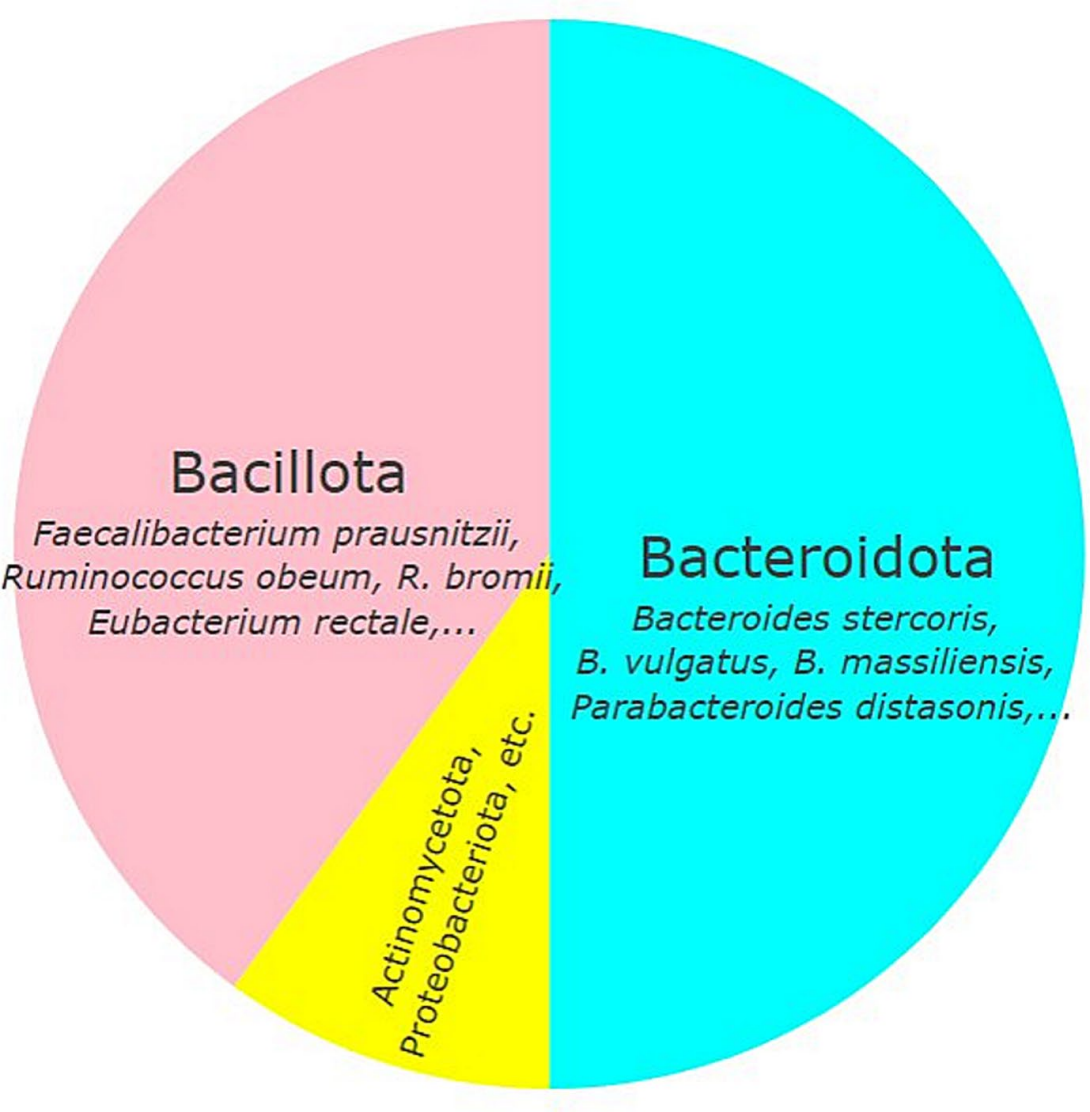
Simplified representation of the relative abundance of gut microbiota phyla in Western populations, and associated species (Tap et al., 2009; Qin et al., 2010; The Human Microbiome Project Consortium, 2012). Created with RStudio (Posit team, 2023), with Plotly.js package v2.11.1 (Sievert, 2020).

The gut microbiota is increasingly recognized as a functionally integrated component of host physiology. Among its major functions are protection from infections (Maciel-Fiuza et al., 2023), shaping of the functionality of the GI tract (Takiishi et al., 2017), body nourishment by digestion of long-chain carbohydrates into short-chain fatty acids (SCFA) (El Kaoutari et al., 2014) and modulation of immune responses (Rooks and Garrett, 2016). In addition, microbiota-derived metabolites can influence distant organs through systemic circulation, contributing to the bidirectional communication network commonly referred to as the gut–brain axis (Wikoff et al., 2009; Carabotti et al., 2015).

The diversity of the microbiota at different body sites is known to have an impact on the host health (Ogunrinola et al., 2020). Gut microbiota composition is influenced by both genetic and environmental factors, including diet (David et al., 2014), birth mode (Dominguez-Bello et al., 2010), lifestyle, and medication exposure (Patterson et al., 2016). Under conditions such as antibiotic treatment, infections, chronic diseases, or major dietary changes, alterations in microbial composition and function may occur, a phenomenon commonly referred to as dysbiosis (Winter and Bäumler, 2023). Dysbiosis is generally characterized by the loss of beneficial commensal microorganisms, the expansion of opportunistic pathogens, or both (Rinninella et al., 2019), and has been associated with several chronic disorders (Iebba et al., 2016; Dabke et al., 2019), including inflammatory bowel disease (Shan et al., 2022), obesity (Liu et al., 2021), diabetes (Gurung et al., 2020), metabolic syndrome (Festi et al., 2014), and neurodegenerative diseases such as Alzheimer’s and Parkinson’s (Sun et al., 2021). Consequently, modulation of the gut microbiota through dietary, probiotic, pharmacological, or fecal microbiota transplantation approaches has emerged as an important area of biomedical research (Zhang Z. et al., 2019; Green et al., 2020).

Because the gut microbiota is highly sensitive to environmental conditions, reproducing its biological behavior *in vitro* requires careful control of the ecological factors that govern microbial community structure and function. Understanding the dynamic interactions between intestinal microorganisms, their metabolites, and the host remains a major scientific challenge (Ma et al., 2024). Recent advances in metagenomics, metabolomics, and transcriptomics have considerably improved the characterization of microbial communities and their functional activity (Gilbert et al., 2018). These technologies have also highlighted substantial interindividual variability in microbiota composition and metabolic responses, contributing to differences in disease susceptibility and responses to dietary or therapeutic interventions (Healey et al., 2017). However, despite these technological advances, studying the gut microbiota remains experimentally difficult due to the complexity of the intestinal ecosystem and the limitations associated with human and animal studies.

These challenges have driven the development of increasingly sophisticated *in vitro* gut microbiota models that reproduce key physicochemical and microbial features of the intestinal environment under controlled conditions. Nevertheless, accurately modeling the gut microbiota extends far beyond simply culturing fecal microorganisms. The biological relevance of an *in vitro* model depends on a series of methodological decisions, including model selection, inoculum preparation, culture medium composition, environmental control, and strategies to maintain microbial stability over time. Collectively, these factors determine how faithfully a model reproduces the native intestinal ecosystem, influences microbial community structure and metabolic activity, and ultimately predicts responses to microbiota-targeted interventions.

In this review, we examine the methodological factors that determine the biological relevance and reproducibility of *in vitro* human gut microbiota models. Previous reviews have extensively described available fermentation platforms and their technical characteristics. Here, rather than providing another catalogue of model types, we adopt a methodological and decision-oriented perspective that considers the experimental workflow as an interconnected system. We examine how model selection, inoculum preparation, culture medium, environmental control, community stabilization, experimental controls and analytical validation collectively determine the interpretation and reproducibility of *in vitro* findings. Biological relevance is therefore considered relative to the biological question being addressed rather than as an intrinsic hierarchy among model types. We further connect these methodological principles to dietary, probiotic and pharmacological applications and discuss how host-integrated, multi-omics and computational approaches can complement conventional fermentation models.

Throughout this review, biological relevance is considered a multidimensional and question-dependent property rather than a single measurable characteristic. For microbiota-focused fermentation models, it may include the capacity to preserve key features of the donor community, maintain representative microbial functions and metabolic outputs, reproduce relevant physicochemical conditions, and generate stable and reproducible responses to experimental perturbations. When host-integrated systems are considered, additional dimensions include epithelial, immune, mechanical and spatial features of intestinal physiology. Importantly, no model is expected to maximize all of these dimensions simultaneously. A model may therefore be biologically relevant for one experimental question while being unsuitable for another.

### 1.1 Literature search

This article was designed as a narrative review informed by a structured literature search rather than as a systematic review or meta-analysis. PubMed was searched initially in 2024 and updated before manuscript submission to include publications available through July 2026. Search terms were combined using Boolean operators and included terms related to the human gut microbiota, *in vitro* fermentation, model design and microbiota-targeted interventions, including “gut microbiota,” “gut microbiome,” “intestinal microbiota,” “fecal microbiota,” “in vitro gut model”, “fermentation”, “batch fermentation”, “continuous culture”, “chemostat”, “gut-on-chip”, “organoid”, “prebiotic”, “probiotic” and “drug.” Peer-reviewed original studies and reviews published in English were prioritized when they addressed the design, validation, operation or application of human gut microbiota models. Particular attention was given to literature published during the preceding 10 years, while earlier seminal studies were retained when they introduced widely adopted models or methodological principles. Studies focusing exclusively on non-gastrointestinal microbiota or pure bacterial cultures without relevance to gut-community modeling were outside the principal scope of the review. Reference lists of selected articles were additionally screened to identify relevant methodological studies that were not retrieved through the primary search.

## 2 Choosing the appropriate *in vitro* model to reproduce the human gut microbiota

The development of an *in vitro* gut microbiota model requires the selection of an experimental system that appropriately reflects the biological processes of interest. Since no model can fully reproduce the complexity of the human intestinal ecosystem, each platform represents a compromise between physiological relevance, ecological stability, experimental throughput, technical complexity, and the host-related processes that can be investigated. Therefore, model selection should be driven by the intended application and the level of biological complexity required.

### 2.1 Comparison of *in vivo* and *in vitro* models

Animal models, particularly mice, have contributed substantially to gut microbiota research by enabling microbial effects to be investigated within an intact host. They have provided important mechanistic insights into interactions between the intestinal microbiota, epithelium and immune system, including in inflammatory bowel disease, where chemical and genetic mouse models have been widely used to investigate the contribution of microbial alterations to intestinal inflammation (Gkouskou et al., 2014). More broadly, findings from human microbiome studies have increasingly been investigated functionally using conventional, genetically modified, or microbiota-associated mouse models to assess the contribution of candidate microbial communities and pathways to disease phenotypes (Basson et al., 2019; Gray et al., 2024). However, mouse models differ substantially from humans in terms of gastrointestinal anatomy and diet, leading to a different gut microbiota composition (Nguyen et al., 2015). Additional differences in physiology, immune function, housing conditions, diet and baseline microbial communities can also influence translation to humans, while animal experimentation entails specific ethical, logistical and economic considerations. Nevertheless, conventional, genetically modified and gnotobiotic mouse models remain important complementary tools for mechanistic microbiota research. Human microbiota-associated mice, for example, can support the engraftment of human fecal communities and reproduce aspects of donor-specific colonization and dietary responses, thereby enabling controlled investigation of how microbial communities influence host biology (Goodman et al., 2011).

Advances in technology have enabled the development of *in vitro* models, such as fermenters and organoids, that closely mimic the human gut environment, hence reducing animal testing (Puschhof et al., 2021; El Houari et al., 2022; Biagini et al., 2023). HGM *in vitro* models are utilized to study food digestion and associated microbiota shifts (Nissen et al., 2020), as well as to explore microbe-microbe interactions and the impact of external factors like dietary compounds or drugs (Isenring et al., 2023). These models may or may not incorporate host interactions, depending on their design and purpose (Biagini et al., 2023). However, most *in vitro* models lack the complexity required to study immune and endocrine responses (Payne et al., 2012). Table 1 lists advantages and disadvantages of using *in vivo* versus *in vitro* models to mimic the HGM.

**Table 1:** Comparison of *in vivo* and *in vitro* models in terms of advantages and disadvantages (Macfarlane and Macfarlane, 2007; Nissen et al., 2020; El Houari et al., 2022; Biagini et al., 2023).

Model type	Advantages	Disadvantages
*In vivo*	Integrates microbiota with whole-organism physiology Enables investigation of epithelial, immune, metabolic and neuroendocrine responses Allows mechanistic perturbation and causal testing Genetically defined and gnotobiotic models permit targeted host–microbe studies Can connect findings from human datasets with experimentally controlled mechanisms	Species-specific differences in gastrointestinal physiology and microbiota Housing, diet, vendor and facility effects may influence results Ethical and animal-welfare considerations Often relatively costly and labor-intensive Throughput generally lower than simple *in vitro* screening systems
*In vitro*	Direct experimental control of microbial and physicochemical variables Allows repeated sampling and controlled perturbations Can isolate direct microbiota responses from host-mediated effects Simple batch/high-throughput platforms can offer relatively rapid and scalable screening Avoids use of experimental animals	Most conventional models lack integrated epithelial, immune, vascular and systemic physiology Culture conditions may impose selective ecological pressures Cost and technical requirements vary substantially between simple batch cultures and sophisticated continuous/multistage systems Human fecal material still requires appropriate ethical approval, informed consent, deidentification/data governance, biosafety and/or biobank oversight Translation to clinical outcomes requires complementary host-based and human evidence

Beyond the distinction between *in vivo* and *in vitro* approaches, gut microbiota models exist along a spectrum of complexity, ranging from simple static batch systems to sophisticated multistage continuous fermentation platforms (Macfarlane and Macfarlane, 2007). Simpler batch systems provide rapid, cost-effective, and high-throughput mechanistic screening, but they poorly reproduce long-term microbial adaptation and ecosystem stability. In contrast, continuous multistage systems improve environmental control, microbial stability, and regional simulation of the gastrointestinal tract, although at the expense of increased operational complexity, reduced scalability, and higher costs (Payne et al., 2012; Venema and Van Den Abbeele, 2013). Despite continuous technological advancements, experimental setups and operating parameters often remain heterogeneous, with no universal consensus regarding optimal model design (Isenring et al., 2023). Consequently, the choice between *in vivo* and *in vitro* approaches depends on the biological question under investigation. While *in vivo* systems remain indispensable for studying host physiology and immune responses, *in vitro* models offer greater experimental control over microbial and physicochemical variables and facilitate repeated sampling and controlled mechanistic investigation of microbial ecology. This review therefore focuses on how these *in vitro* systems can be optimized to maximize their biological relevance. The following sections examine the major *in vitro* fermentation systems used for gut microbiota research and discuss how their design and operational parameters influence microbial stability, reproducibility, and biological relevance.

### 2.2 Batch fermentation systems (static)

Batch fermentation systems represent the simplest approach for reproducing the gut microbiota *in vitro* and are generally selected when short-term microbial responses are the primary objective. Their principal strength lies in providing a controlled environment for rapid mechanistic studies, although their biological relevance progressively decreases during prolonged incubation (Nissen et al., 2020). These models are typically set up in well plates, closed test tubes or bioreactors (Biagini et al., 2023). The latter allows precise control through feedback mechanisms that regulate pH, oxygen levels, temperature, and mixing, thereby enabling tighter environmental control than other static fermentation systems (Nissen et al., 2020; Isenring et al., 2023). Operating in a closed environment, these models specifically simulate one portion of the gastrointestinal tract (Biagini et al., 2023). The microbial biomass, cultivated in a defined medium, consists of a single-cell type or complex microbial communities. In such systems, microbial growth is primarily influenced by the inoculum density (the initial concentration of microbes) and the substrate depletion rate (the speed at which nutrients are consumed) (Payne et al., 2012; Sardelli et al., 2021).

However, the closed nature of batch systems makes it challenging to monitor changes in microbiota composition after inoculation (Macfarlane and Macfarlane, 2007), while repeated sampling may itself disturb fermentation conditions. Additionally, the incubation period is commonly limited to 24–48 h due to nutrient exhaustion and toxic metabolite accumulation (Isenring et al., 2023), which can disrupt microbial balance (Nissen et al., 2020). Beyond these immediate constraints, the lack of continuous nutrient supply and metabolite removal promotes time-dependent ecological drift, defined as progressive culture-induced deviations from the original microbial community resulting from selective pressures imposed by the *in vitro* environment. This process often favours fast-growing, metabolically flexible taxa while disadvantaging oxygen-sensitive or nutritionally fastidious organisms. Hence, rapid changes in media acidity, redox potential and community structure (Venema and Van Den Abbeele, 2013) progressively reduce the ecological and functional fidelity of batch models to the original microbial community (Gibson and Fuller, 2000; El Houari et al., 2022). As a consequence, batch systems are mainly suitable for initial screening assays and the study of acute microbial responses, such as bacterial adhesion and infection (Sardelli et al., 2021), or the interaction of specific compounds with gut microbial communities (Nissen et al., 2020), including their metabolization or digestion (Payne et al., 2012; Venema and Van Den Abbeele, 2013). Finally, batch models are not appropriate for investigating chronic phenomena such as gut inflammation.

The following three short examples highlight the versatility of batch models in elucidating specific microbial responses and interactions within the gut environment. (i) In a medium containing mucin to mimic the gut environment, fermented feces showed an increased abundance of *Escherichia coli*, members of the order Enterobacterales, and other taxa (Raimondi et al., 2021). (ii) Under more acidic conditions (e.g., decreased pH resulting from substrate fermentation), an increase in the abundance of butyrate-producing bacteria and a decrease of *Bacteroides* spp. in the culture medium have been demonstrated (Walker et al., 2005). (iii) *In vitro*, trans-galactooligosaccharides and inulin have demonstrated prebiotic effects, defined as the selective stimulation of beneficial microorganisms, leading to an increased abundance of *Bifidobacterium* spp., which are associated with the regulation of energy homeostasis and body weight (Vulevic et al., 2013). Collectively, these examples illustrate that batch fermentation systems can capture rapid microbial responses to environmental perturbations, including nutrient availability, pH variation, and substrate supplementation. However, because these responses occur within an evolving ecosystem lacking continuous nutrient renewal, they should primarily be interpreted as short-term ecological adaptations rather than stable representations of the *in vivo* gut microbiota.

Transitioning from batch to fed-batch conditions represents an intermediate strategy to improve microbial maintenance and metabolic stability. In fed-batch systems, fresh medium is periodically supplied to reduce nutrient depletion and partially limit toxic metabolite accumulation, thereby prolonging microbial activity compared with fully static cultures. Nevertheless, because waste products are not continuously removed, these systems remain susceptible to ecological drift and progressive alterations in microbial composition over time. Dynamic continuous fermentation systems address these limitations more effectively by incorporating simultaneous medium inflow and outflow, thereby maintaining more stable environmental conditions and prolonged culture viability (cf. Figure 2) (Sardelli et al., 2021). Fed-batch systems therefore provide an intermediate operational strategy when prolonged microbial activity is required without implementing continuous-flow fermentation.

**Figure 2:**
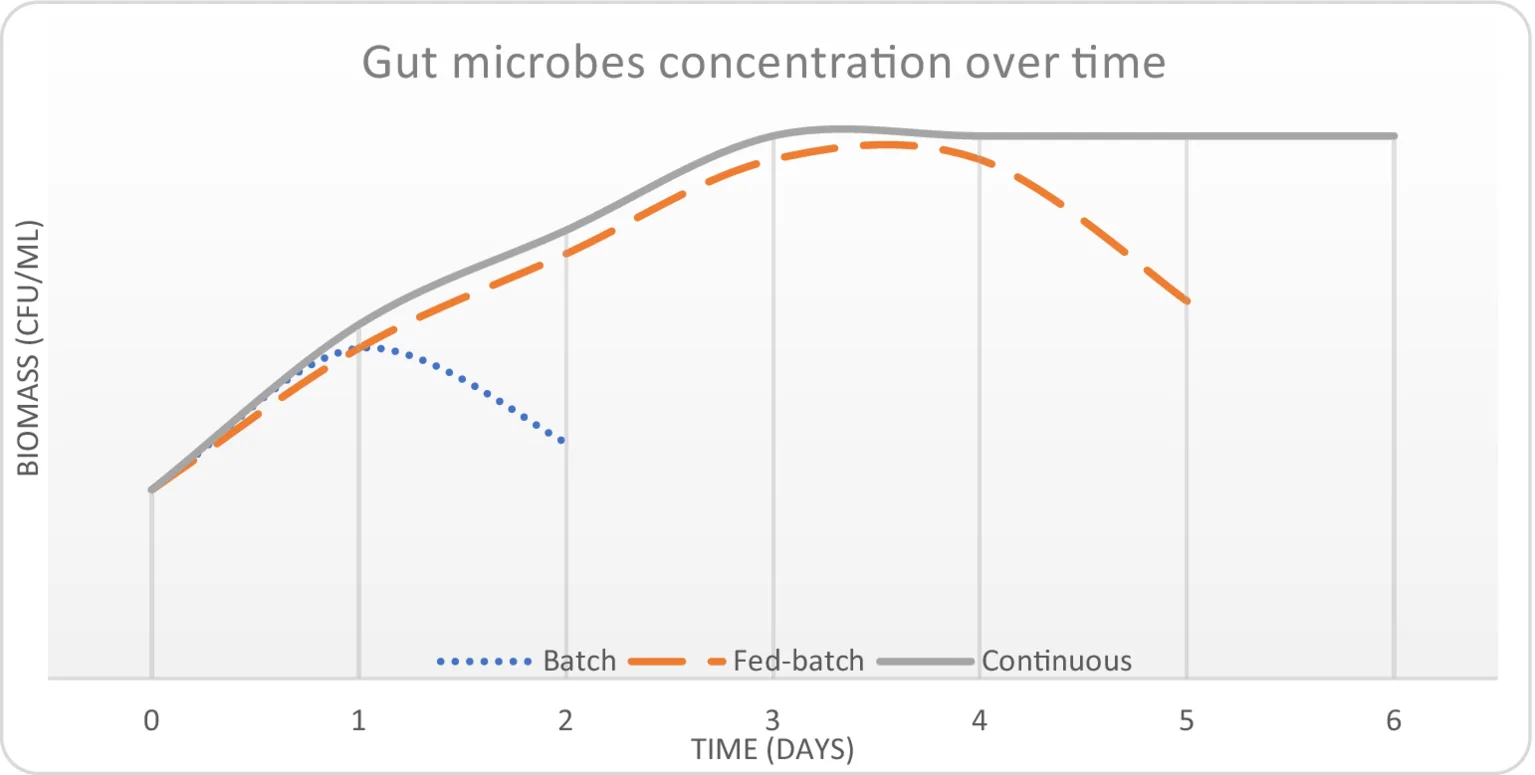
Conceptual representation of expected biomass trajectories in batch, fed-batch and continuous fermentation systems.

### 2.3 Continuous culture systems (dynamic)

When prolonged maintenance of microbial communities under controlled nutrient supply and waste removal is required, continuous culture systems provide important advantages over closed batch cultures. In continuous-flow systems, fresh culture medium is supplied while an equivalent volume of culture is removed, thereby maintaining an approximately constant culture volume. A chemostat represents a specific type of continuous culture in which microbial growth is controlled by the dilution rate and the availability of a growth-limiting nutrient. In gut microbiota research, dynamic fermentation platforms may employ continuous-flow, semi-continuous, or compartment-specific feeding strategies and should therefore not all be considered classical chemostats (Isenring et al., 2023). Continuous medium exchange enables nutrient replenishment and purging of newly-produced toxic metabolites, keeping the microbial balance over an extended period (Payne et al., 2012; Nissen et al., 2020) and facilitating the establishment of steady-state conditions (Poeker et al., 2019). Due to the complexity of monitoring dynamic systems (refer to chapter “culture maintenance and challenges” of this review), dynamic models are equipped with various probes and ports for real-time analysis (Biagini et al., 2023). These systems are particularly well suited to prolonged experiments investigating microbial adaptation and sustained responses to prebiotics, probiotics, dietary compounds, or other repeated perturbations (Venema and Van Den Abbeele, 2013; El Houari et al., 2022).

Dynamic continuous fermentation systems can be configured as single-stage or multistage platforms. Complexity increases with the number of successive compartments in the model (Payne et al., 2012), because each compartment has its own specifically configured parameters (e.g., pH, oxygen, temperature and transit time) to mimic different sections of the digestive tract (Biagini et al., 2023). In mono-compartmental models, such as the artificial colon that reproduces only the colon environment, it has been possible to study the metabolization of secoisolariciresinol diglucoside, whose metabolites have shown health benefits (Quartieri et al., 2016), and of chlorogenic acid, a phenolic acid abundant in the Western diet (Tomas-Barberan et al., 2014). Single-stage models, including twin-vessel systems, have demonstrated their ability to develop and maintain stable, diverse, and reproducible microbial communities (McDonald et al., 2013). Multi-stage systems require more advanced control systems and greater technical and operational resources than single- or two-stage bioreactors (Sardelli et al., 2021). One of the first examples is the Reading Model, developed by Gibson and Macfarlane in 1988 and made up of three consecutive vessels which mimic the proximal, transverse and distal colon (Gibson et al., 1988). A few years later, the TNO gastro-intestinal model (TIM) was created and comprises four compartments mimicking the conditions of stomach, duodenum, jejunum and ileum (Minekus, 2015). Other examples are the EnteroMix (Makivuokko et al., 2005), the Lacroix model (Cinquin et al., 2004) and the SHIME**®** (Simulator of the Human Intestinal Microbial Ecosystem) (Van Den Abbeele et al., 2010). In the latter, pancreatic and bile juices are pumped to some vessels at precise intervals (Nissen et al., 2020). The addition of host digestive functions helped to develop artificial digestive systems, enhancing physiological relevance and representing the most advanced fermentation modeling (Payne et al., 2012). *Bifidobacterium* growth and SCFA production have been largely studied by using those models (Venema and Van Den Abbeele, 2013). Continuous multistage systems can reproduce region-specific physicochemical conditions and therefore offer important advantages when gastrointestinal spatial organization and prolonged microbial adaptation are central to the research question. However, these advantages come at the expense of greater technical complexity, cost and reduced throughput, and they do not necessarily make multistage systems the optimal choice for all applications.

A summary of the main characteristics, advantages, limitations, and applications of the different fermentation models discussed in this section is provided in Table 2.

**Table 2:** Comparison of major gut microbiota fermentation models according to their main characteristics and applications (Macfarlane and Macfarlane, 2007; Nissen et al., 2020; El Houari et al., 2022; Biagini et al., 2023).

Parameter	Batch	Fed-batch	Single-stage continuous	Multistage continuous
Experimental design	Closed system; no nutrient renewal or waste removal	Periodic nutrient addition; limited waste accumulation control	Continuous nutrient supply and waste removal	Multiple interconnected vessels reproducing different gastrointestinal regions
Complexity, cost and capacity for prolonged microbial stability	Low	Moderate	High	Very high
Main applications	Rapid screening, acute microbial responses, substrate or compound testing	Intermediate-term studies requiring extended microbial activity	Long-term studies of microbial metabolism, adaptation, probiotics and prebiotics	Complex dietary interventions, gastrointestinal simulation, and ecosystem-level studies

## 3 Optimizing the microbial inoculum

### 3.1 Inoculum selection, preparation, and preservation

Once an appropriate fermentation system has been selected, preserving the biological relevance of the model largely depends on the quality of the microbial inoculum (i.e., the microbial material introduced into the culture system to initiate fermentation). The composition, viability, and handling of the inoculum determine the initial microbial ecosystem introduced into the reactor and therefore strongly influence downstream fermentation behavior (Biagini et al., 2023). Inocula may consist of defined bacterial strains (El Houari et al., 2022), fecal samples from healthy volunteers or patients, or a combination of both, depending on the experimental objective and the desired level of complexity. When using human feces, approval from a research ethics committee is mandatory (Raimondi et al., 2021), as informed written consent from donors alone may not be sufficient (Dostal et al., 2015). Donors should not have received antibiotics for at least three months, and prebiotics or probiotics for at least one month prior to sample collection (Tomas-Barberan et al., 2014; Jones et al., 2021; Raimondi et al., 2021). Other donor-related variables such as diet, metabolic status, age, BMI, sex, and gastrointestinal health should be considered, as these factors significantly influence baseline microbial composition and functional potential (Hasan and Yang, 2019).

From a biological perspective, sample collection and handling represent one of the greatest sources of experimental bias because they directly influence the survival of oxygen-sensitive microorganisms. Best-practice guidelines therefore recommend minimizing oxygen exposure and processing samples as rapidly as possible to preserve the original microbial community (Widjaja and Rietjens, 2023). Fecal material should therefore be collected under sterile conditions, using a stool collector (e.g., Fecotainer®) or a screw-capped container. Once collected, samples should be placed in resealable bags for secondary containment and maintained at 4 °C during transport (Pérez-Burillo et al., 2021).

The optimal storage strategy depends on the downstream objective. Conditions that adequately preserve DNA or broad taxonomic profiles for sequencing do not necessarily preserve microbial viability or metabolic activity to the same extent. For samples intended as inocula for *in vitro* fermentation, rapid processing under chilled and oxygen-limited conditions is therefore preferable whenever feasible. When immediate use is not possible, validated cryopreservation procedures, typically involving aliquoting and storage at −80 °C, may be used to enable subsequent fermentation experiments (Jones et al., 2021). Samples are commonly weighed individually upon receipt (Pérez-Burillo et al., 2021).

Although some processing steps may be performed under ambient conditions for practical reasons, prolonged oxygen exposure should be minimized because it can preferentially reduce strict anaerobes (Burz et al., 2019). Accordingly, fresh samples should be sealed in anaerobic plastic bags and handled under oxygen-limited conditions prior to *in vitro* fermentation (Raimondi et al., 2021). The preparation of the inoculum is hence typically performed in an anaerobic cabinet under an atmosphere of approximately 85% N_2_, 10% CO_2_ and 5% H_2_ (Tomas-Barberan et al., 2014; Raimondi et al., 2021).

Fecal samples used as inoculum can be introduced in different forms: as liquid suspensions without any physical substrate, or immobilized within polymer beads. For liquid forms, feces are usually homogenized with either the culture medium or PBS buffer (containing a reductive agent such as L-cysteine hydrochloride to maintain low redox potential) at a ratio of 5–10% (w/v) (Tomas-Barberan et al., 2014; Quartieri et al., 2016). Alternatively, microorganisms can be immobilized in gel beads (e.g., using 1–2 mm gel beads composed of gellan gum, xanthan gum, and sodium citrate), which support stable bacterial diversity for extended periods of time (Lacroix et al., 1990; Lamboley et al., 1997; Cinquin et al., 2006). Micro-encapsulation has also been used as an advanced form of inoculum delivery (Zihler et al., 2011). All immobilization procedures are completed under aseptic conditions (Isenring et al., 2023). The structure of the inoculum strongly influences community resilience and stability: systems employing liquid fecal inocula are more prone to rapid compositional shifts and the elimination of less competitive bacteria due to competitive growth dynamics (Payne et al., 2012), whereas immobilized inocula tend to better preserve low-abundance and fastidious microorganisms over time. Consequently, the choice of inoculum format should be guided by the intended application: liquid inocula are generally appropriate for short-term mechanistic studies, whereas immobilized systems may be advantageous for prolonged experiments in which maintenance of community diversity and less competitive taxa is important.

The inoculum density is a critical factor influencing microbial growth, community development, and metabolic activity in fermentation systems (Sardelli et al., 2021). Rather than being a fixed parameter, the optimal inoculation ratio depends on the experimental design, culture conditions, and study objectives. For instance, low inoculum ratios (0.1–1%) have been used to investigate microbial growth dynamics and community adaptation, as they allow greater microbial expansion during fermentation (Monteagudo-Mera et al., 2023). Conversely, higher inoculum ratios (>10%, w/v) have been employed in fermentation models focused on substrate degradation and metabolite production, where microbial biomass primarily provides enzymatic and metabolic capacity rather than supporting extensive growth (Aura, 2005). Intermediate inoculum concentrations have also been reported in gut microbiota models, with fecal inocula around 3% supporting representative growth conditions of the human intestinal microbiota (Kim et al., 2011). Therefore, inoculum density should be experimentally optimized, with the selected ratio ideally preserving a microbial community composition as close as possible to the original inoculum after the adaptation period. Indeed, following inoculation, microbial communities may undergo adaptation to reactor conditions, during which selective pressures imposed by the *in vitro* environment progressively reshape community structure (Nissen et al., 2020).

Substantial interindividual variability exists even among healthy donors, resulting in marked differences in microbial diversity, taxonomic composition, and metabolic activity (Healey et al., 2017). Consequently, fecal microbiota pooling is frequently used in *in vitro* fermentation studies to reduce donor-to-donor variability and improve the representativeness and reproducibility of experimental outcomes. However, pooling samples may also generate artificial community interactions and mask donor-specific microbial signatures, limiting its applicability for mechanistic studies or personalised microbiota research. In contrast, individual donor inocula better preserve subject-specific microbial characteristics, although they introduce greater biological variability between experiments (Pérez-Burillo et al., 2021; Isenring et al., 2023). Therefore, the choice between pooled and individual donor samples represents a balance between experimental reproducibility and preservation of donor-specific biological features.

Finally, for longer-term storage and reproducibility studies, fecal slurries are commonly aliquoted and frozen at −80 °C until use (Allegretti et al., 2020; Bottino et al., 2024). This approach enables repeated experiments using the same donor microbiota and allows simultaneous inoculation of multiple reactors with a standardized microbial starting material, thereby improving inter-experimental comparability (Tomas-Barberan et al., 2014). The extent to which strict anaerobic conditions are required during storage depends on the microorganisms and functional properties of interest; nevertheless, limiting oxygen exposure is advisable when preservation of highly oxygen-sensitive taxa is important (Burz et al., 2019). To reduce osmotic and mechanical stress on bacteria during the freezing process, a cryoprotective agent (e.g., glycerol or inulin) can be added (Bircher et al., 2018b; Bilinski et al., 2022; Isenring et al., 2023), but it should ideally be removed prior to inoculation to avoid transient effects on microbial metabolism (De Carvalho et al., 2021). Importantly, although frozen inocula improve experimental reproducibility across time, they may not fully preserve the viability, functional activity, and metabolic state of the original microbial community. Freeze–thaw cycles can also differentially affect microbial viability, with strict anaerobes generally being more sensitive than facultative organisms (Bircher et al., 2018a; Sangermani et al., 2026). Alternatively, reproducibility can be improved by inoculating several reactors simultaneously using the same fresh fecal sample (Isenring et al., 2023). Overall, storage conditions should be viewed as an integral component of model design, as they directly influence microbial viability, functional activity, and ultimately the biological relevance and reproducibility of the fermentation model. Beyond donor-to-donor variability in bacterial composition, the biological characteristics of an inoculum also depend on the representation of non-bacterial microbial groups and on whether luminal or mucus-associated communities are captured.

### 3.2 Multi-kingdom and spatial representation of the gut microbiota

Although bacteria remain the most extensively characterized component of the gut microbiota and are the primary focus of most *in vitro* fermentation studies, the intestinal ecosystem is intrinsically multi-kingdom. In addition to bacteria, it contains archaea, fungi, bacteriophages and other viruses, protozoa, and other microbial eukaryotes that participate in ecological interactions within the gut environment (Garcia-Bonete et al., 2023; Chen et al., 2024). These organisms can influence community structure and function through mechanisms including competition for nutrients, metabolic cross-feeding, modulation of bacterial metabolism, and, in the case of bacteriophages, predation of bacterial hosts (Ventura et al., 2011; Liu et al., 2025). Consequently, preservation of bacterial diversity alone does not necessarily indicate that the broader ecological complexity of the donor community has been maintained.

The extent to which conventional *in vitro* fermentation systems preserve non-bacterial community members remains insufficiently characterized. Most gut fermentation studies have historically focused on bacterial communities, and model validation is commonly based on bacterial 16S rRNA gene profiling and bacterial metabolic output (Isenring et al., 2023). Fungi and intestinal protozoa may require specific culture conditions that differ from those used for bacterial communities, including distinct media, redox conditions, or other growth requirements (Clark and Diamond, 2002; Nilsson et al., 2019). Bacteriophage dynamics may also change as bacterial host populations and physiological states shift during fermentation, given the strong dependence of phage ecology on bacterial host availability and growth dynamics (Mirzaei and Maurice, 2017). Assessment of multi-kingdom community preservation therefore requires analytical approaches extending beyond bacterial 16S rRNA gene profiling, including, depending on the organisms of interest, shotgun metagenomics, ITS sequencing, viromics, microscopy, or targeted molecular methods.

Donor-screening procedures represent an additional consideration. In clinical fecal microbiota transplantation programs, detection of intestinal protozoa such as *Blastocystis* spp. or *Dientamoeba fragilis* has frequently been used as a criterion for donor exclusion, although screening protocols vary considerably and the clinical significance of asymptomatic carriage remains debated (Bénard et al., 2022). Importantly, carriage of some intestinal protozoa has also been associated with distinct bacterial community structures and, in some studies, greater bacterial diversity (Audebert et al., 2016). Therefore, systematic exclusion of donors carrying particular protozoa or other microeukaryotes may unintentionally restrict the range of microbial community configurations represented by fecal inocula. Although donor selection for experimental fermentation studies serves a different purpose from clinical FMT screening, the screening criteria applied should be transparently reported and aligned with the biological question being investigated.

Spatial organization provides a further dimension of intestinal microbial complexity. Fecal inocula predominantly reflect luminal and distal intestinal communities and do not fully capture microorganisms associated with the intestinal mucus layer and epithelium (Delbaere et al., 2023). Mucus-associated microorganisms experience distinct nutrient availability, oxygen exposure, host-secreted compounds, and spatial interactions compared with luminal communities (Calvigioni et al., 2023). Consequently, models relying exclusively on fecal suspensions may underrepresent microbial populations adapted to the mucosal niche. Some advanced fermentation systems address this limitation through mucin-containing media, mucin-coated carriers, or dedicated mucosal compartments that permit colonization by mucus-associated microorganisms (Vermeiren et al., 2012; Calvigioni et al., 2024). Nevertheless, these approaches remain approximations of the continuously renewed and host-regulated intestinal mucus environment.

## 4 Culture media: building a biologically relevant gut environment

Following inoculum selection, the culture medium becomes one of the principal determinants of an *in vitro* gut microbiota model. The medium must not only sustain microbial survival but also preserve the metabolic interactions and ecological balance that characterize the native intestinal ecosystem (Sardelli et al., 2021). While various media already exist, comprising both common and specific nutritional compounds, no single formulation is universally accepted for *in vitro* HGM modeling. The composition of the culture medium profoundly influences both microbial community structure and metabolic output, including SCFA and other key metabolites, by selectively shaping taxonomic composition through variations in carbon and nitrogen availability, mucin supplementation, bile salts, and redox conditions (Biagini et al., 2023). Medium composition should therefore be considered a major determinant of biological relevance rather than merely a technical parameter (Payne et al., 2012). Consequently, differences in medium formulation may reduce reproducibility and comparability between studies, contributing to inconsistent taxonomic and functional outcomes across *in vitro* HGM models (McDonald et al., 2013).

In designing a medium, its composition should ideally mimic the chyme entering the colon, meaning that it may need to be adapted to the diet of the target population (Isenring et al., 2023). Consequently, medium formulation should be selected according to the biological question being investigated. Two distinct trophic approaches have been described in fermentation systems. Oligotrophic media, inoculated with a high concentration (5–20% w/v) of fecal slurry and supplemented with minimal nutrients, aim to reflect the gut’s status under normal or calorie-restricted diets. In contrast, eutrophic media are typically inoculated with around 1% fecal slurry into a basal medium enriched with peptone, yeast extract and bile salts (Long et al., 2015), where the higher peptide content can lead to increased ammonia production that better reflects *in vivo* conditions (Walker et al., 2005). Eutrophic media hence simulate the nutrient-rich environment associated with high-fat and high-protein diets. Using both trophic approaches may be informative when evaluating the modulatory effects of a potential prebiotic substrate on HGM *in vitro* models (Long et al., 2015). In practice, although media such as brain heart infusion broth, high/low concentration carbohydrate media, or fastidious anaerobe broth can be used (Biagini et al., 2023), enriched culture media are usually preferred for maintaining complex communities (Bonnet et al., 2020). For batch fermentations, a minimal medium can help to highlight the metabolism of the compound or dietary component under investigation and facilitate fast identification of the resulting metabolites or involved bacteria. Conversely, in continuous culture systems, a rich medium is often advantageous because it stabilizes the microbial community prior to introducing the test substance (Pérez-Burillo et al., 2021). The choice between minimal and enriched media therefore represents a compromise between mechanistic specificity and ecological fidelity.

The composition of culture media directly determines the metabolic landscape experienced by the microbial community. Consequently, each nutritional component acts as a selective ecological factor influencing bacterial competition, metabolite production, and community stability. Most *in vitro* media formulations combine carbon sources (e.g., yeast extract, starch, glucose), nitrogen sources (e.g., casein hydrolysate, tryptone) and mineral salts (e.g., phosphate, calcium, magnesium). Additional elements such as amino acids or vitamins can also be incorporated (Biagini et al., 2023). Water dissolves and transports the nutrients and is required for hydrolysis of reactants. The nature and complexity of carbon substrates strongly influence saccharolytic fermentation pathways and SCFA production, whereas peptide-rich nitrogen sources can favor proteolytic metabolism and increased ammonia generation (Procházková et al., 2023). Certain fastidious bacteria require specific growth factors that they cannot synthesise from the nutrients present in the medium: vitamins, nitrogenous bases and heme sources (Bonnet et al., 2020). To study the mucosal compartment, some models incorporate mucin into the medium (De Boever et al., 2000; Calvigioni et al., 2023), providing insights into how colonic bacteria break down this glycoprotein under conditions of both health and disease (e.g., IBD) (Venema and Van Den Abbeele, 2013). Figure 3 illustrates the occurrence of compounds present in ten different media used for gut microbiota modeling. Notably, only seven compounds are found in more than half of the media, highlighting the lack of standardization in the field.

**Figure 3:**
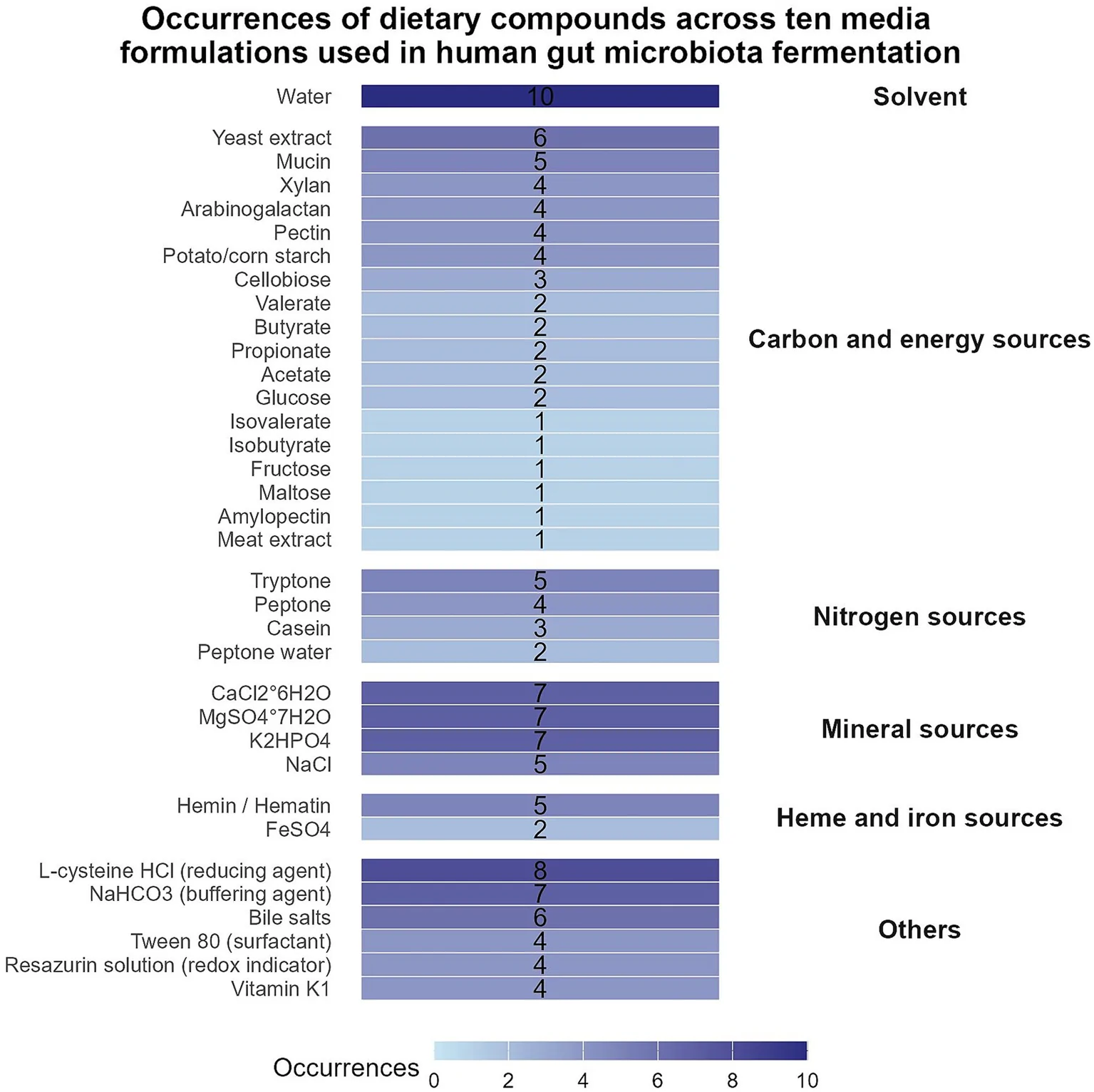
Occurrence of the compounds found in ten different media used for human gut microbiota modeling (De Boever et al., 2000; Roowi et al., 2010; Goodman et al., 2011; McDonald et al., 2013; Khalil et al., 2014; Quartieri et al., 2016; Zhang X. et al., 2019; Navarro Del Hierro et al., 2020; Raimondi et al., 2021; Cui et al., 2023). Created with RStudio (Posit team, 2023), with ggplot2 package v4.0.3 (Wickham et al., 2007).

Collectively, recent studies demonstrate that relatively small variations in medium composition can profoundly alter both microbial community structure and metabolic activity. Low-carbohydrate media significantly reduce microbial biodiversity and SCFA production, whereas commercial Schaedler broth supplemented with 5% sheep blood helps to preserve both high biodiversity and metabolic activity (Średnicka et al., 2023). In another study, four media were compared and it was found that variations in sugar, vitamin, and mucin content significantly influenced the production of acetic, propionic, and butyric acids, with media containing prebiotics like inulin (BGM media) promoting the highest butyrate levels (Yousi et al., 2019). Additionally, efforts to design several defined media to accommodate the diverse nutritional requirements of 96 gut bacterial strains have demonstrated that while many species grow robustly in defined media, the presence or absence of SCFAs and mucin can markedly alter growth patterns (Tramontano et al., 2018).

Overall, culture media should be regarded as a central component of *in vitro* model design rather than a simple nutritional support. Their formulation determines microbial selection, metabolic activity, ecological stability, and ultimately the biological relevance of the experimental system. Future efforts should therefore focus on developing standardized yet biologically representative media capable of preserving the complexity and functionality of the native gut microbiota while improving reproducibility across laboratories.

## 5 Culture maintenance and challenges

Selecting an appropriate fermentation system, inoculum, and culture medium alone is insufficient to ensure a biologically relevant *in vitro* model. Once fermentation has begun, maintaining environmental conditions that preserve microbial ecology becomes equally critical. Because the *in vitro* environment lacks many of the regulatory mechanisms present in the human gut, continuous monitoring and control of culture parameters are required to minimize ecological drift and preserve physiological relevance throughout the experiment (Sardelli et al., 2021).

### 5.1 Environmental control and key parameters for monitoring

Maintaining biological relevance during fermentation requires reproducing the environmental conditions that naturally shape microbial ecology within the human gastrointestinal tract: pH, partial oxygen pressure, redox potential, transit time, nutrient supply and host secretions (Biagini et al., 2023). These variables act as primary ecological filters that determine which taxa persist, proliferate, or decline under given conditions. Moreover, establishing the correct initial conditions is important to prevent the overgrowth of fast-growing bacteria and the underrepresentation of more sensitive species. Because these parameters vary substantially among individuals, defining representative or selecting median conditions (based on age, diet, and overall health) is crucial, although it remains challenging given the limited *in vivo* data (Isenring et al., 2023).

For batch fermenters, temperature, pH and anaerobiosis state are the primary controlled variables (Payne et al., 2012). The temperature is typically maintained at 37 °C using a thermal jacket or a water bath, while pH is generally maintained within a near-neutral range (approximately 6.8–7.2) to reflect colonic conditions. pH is one of the most influential regulators of gut microbial ecology, as it directly affects enzymatic activity, substrate utilization, and interspecies competition (Xie et al., 2024). Note that substrate fermentation naturally lowers the pH, which can boost butyrate production and promote butyrate-producing bacteria while inhibiting the growth of certain taxa (e.g., Bacteroides spp.) (Walker et al., 2005). In one study, cultures initiated at a mean pH of 6.9, compared to those at 7.6, exhibited a reduced shift in community composition (Rachmühl et al., 2023). Regular monitoring using calibrated probes ensures that the fermenter remains at the desired pH and temperature. To maintain the fermenter at a relatively neutral pH, solutions of HCl or NaOH are usually delivered (Isenring et al., 2023). These feedback systems are essential to prevent uncontrolled ecological drift. The bacterial composition is also strongly influenced by the oxygen concentration, as many dominant gut microorganisms are obligate anaerobes whose metabolic pathways depend on strictly reducing conditions. Oxygen exposure can therefore disrupt community structure by suppressing oxygen-sensitive taxa and favoring facultative anaerobes. For this reason, anaerobic gas mixtures (typically N₂/CO₂/H₂) are used to maintain reducing conditions during fermentation (Sardelli et al., 2021), with nitrogen often preferred to avoid CO₂-driven pH fluctuations (Nissen et al., 2020). Collectively, these parameters determine whether the microbial community remains representative of the original inoculum or progressively diverges through culture-induced ecological drift. Their continuous control is therefore fundamental to preserving biological relevance throughout batch fermentation.

For continuous fermenters, in addition to temperature, pH, and anaerobiosis, monitoring includes hydraulic retention time and flow rate (Payne et al., 2012), enabling a more physiologically relevant simulation of colonic conditions. A constant medium flow prevents nutrient depletion, accumulation of putrefactive metabolites and the expression of enzymes involved in the formation of genotoxic compounds. This steady flow stabilizes the microbial community over extended periods (Venema and Van Den Abbeele, 2013).

### 5.2 Community stability assessment

Community stability represents one of the principal indicators that an *in vitro* model has successfully preserved the biological characteristics of the original gut microbiota. In continuous or prolonged culture systems, stability is typically evaluated over periods exceeding 1 week by combining microbial community analysis with metabolic activity measurements (Galloway-Peña and Hanson, 2020). For example, 16S rRNA gene amplicon sequencing or metagenomics can be used to track community composition, since they generate relative abundance data (Galloway-Peña and Hanson, 2020; Isenring et al., 2023). In practice, microbial stability is commonly assessed by comparing the temporal similarity of the community to the initial inoculum, using ecological distance metrics such as Bray–Curtis dissimilarity (Pérez-Burillo et al., 2021; Jin et al., 2022). Because relative abundance does not reflect absolute population size, combining these approaches with qPCR or flow cytometry allows absolute quantification of specific taxa and total bacterial load, providing a more accurate interpretation of true compositional changes (Jian et al., 2020). Combining taxonomic and quantitative approaches therefore provides a more comprehensive assessment of biological stability than either method alone.

The production of short-chain fatty acids (SCFAs) serves as a rapid indicator of metabolic function (Isenring et al., 2023). The proportion of key SCFAs in a culture should closely approximate that observed in fecal samples, despite higher absolute concentrations *in vitro* (Zihler Berner et al., 2013). The main SCFAs produced by gut microbial fermentation are acetate, propionate, and butyrate, which are typically found in the colon at an approximate molar ratio of 60:20:20, respectively (Cummings, 1981). Importantly, SCFA profiles should be interpreted as functional readouts rather than direct proxies of taxonomic stability, as metabolic stability may be maintained even when compositional shifts occur (Louca et al., 2018). In studies designed around stabilized communities, interventions should be introduced only after predefined stability criteria have been met. Another indicator of stability is biomass production, which can be monitored by measuring the optical density of the culture medium as a proxy for overall microbial growth and viability (Isenring et al., 2023). Together, compositional, quantitative and functional analyses provide complementary evidence that a fermentation model has maintained biological relevance throughout the experiment.

Recent investigations into batch fermentation systems illustrate the challenges in achieving stability. For instance, one study demonstrated that although the top abundant genera of Kenyan infants’ fecal microbiota regrew after transport and 24-h batch cultivation, the relative abundances differed from the original fecal samples (Rachmühl et al., 2023).

### 5.3 Experimental controls

The use of both baseline and negative controls is essential for correctly interpreting microbiota modulation experiments. Baseline controls correspond to the initial microbial composition prior to fermentation and are used to assess intrinsic community changes over time. In contrast, negative controls consist of parallel fermentations performed without the tested intervention, allowing discrimination between spontaneous fermentation-driven shifts and treatment-specific effects. This dual-control strategy prevents misinterpretation of treatment effects. For example, in one study, a prebiotic formula showed no significant effect on predominant *Faecalibacterium* species when compared with baseline samples; however, when assessed against untreated negative controls, the same formula stimulated these beneficial bacteria. Such results underscore the need for appropriate control strategies when evaluating the modulatory effects of dietary interventions (Long et al., 2015). Proper controls therefore increase confidence that observed microbial changes result from the tested intervention rather than the fermentation process itself. When appropriate, positive controls using compounds or interventions with well-established microbiota-modulating effects may also be included to verify the responsiveness of the experimental system.

### 5.4 Reproducibility best practices

An *in vitro* model is considered reliable when it consistently reproduces the key characteristics of the *in vivo* gut microbiota across independent experiments. Reproducibility can be demonstrated by running multiple experiments using fecal slurries from the same donor at different times (to assess consistency in microbial composition and metabolic outputs), as well as by comparing results across samples from different donors (to evaluate the model robustness and generalizability) (McDonald et al., 2013; Biagini et al., 2023).

Enhancing model stability is essential and depends on both the precise control of culture parameters and the selection of an appropriate model, which should be tailored to the specific research question. While there is an inherent compromise between technical complexity and biological relevance, steady-state conditions must be strictly maintained to achieve reproducibility (Payne et al., 2012). Ultimately, replicates from both identical and distinct microbiota sources increase the confidence and validity of the model, while also providing insights into shared features among individuals that may inform personalized microbiota modulation strategies (Isenring et al., 2023).

Collectively, these considerations demonstrate that achieving biologically relevant and reproducible *in vitro* gut microbiota models depends on careful methodological planning at every stage of the experimental workflow. Table 3 summarizes the most common methodological pitfalls, their potential consequences, and practical recommendations to improve experimental reliability.

**Table 3:** Common methodological pitfalls encountered during the design and operation of in vitro human gut microbiota models, together with their potential consequences and recommended best practices.

Methodological aspect	Common pitfall	Potential consequence	Recommended best practice
Model selection	Selecting a fermentation model that does not match the experimental objective	Reduced biological relevance or unnecessary technical complexity	Define the biological question first, then select the simplest model capable of answering it
Donor selection	Inadequate donor characterization or recent antibiotic/probiotic use	Altered baseline microbiota and poor reproducibility	Apply standardized donor inclusion criteria and report donor characteristics
Sample handling	Delayed processing or prolonged oxygen exposure	Loss of strict anaerobes and altered community composition	Minimize oxygen exposure, transport at 4°C, and process samples rapidly under anaerobic conditions
Inoculum preparation	Non-standardized inoculum density or preparation protocol	Variable microbial growth and inconsistent metabolic activity	Optimize inoculum concentration according to the experimental objective and standardize preparation procedures
Frozen inocula	Repeated freeze–thaw cycles or inappropriate cryopreservation	Reduced viability and altered microbial functionality	Store aliquots at −80 °C, avoid repeated thawing, and use validated cryoprotective agents
Culture medium	Medium composition not adapted to the research objective	Selective enrichment or depletion of microbial taxa and altered metabolite production	Match medium composition to the intended biological question and report all components in detail
Environmental control	Poor regulation of pH, temperature or anaerobiosis	Ecological drift and overgrowth of facultative anaerobes	Continuously monitor temperature, pH and anaerobic conditions throughout fermentation
Continuous systems	Incorrect flow rate or hydraulic retention time	Nutrient depletion or unstable microbial communities	Optimize flow parameters according to reactor design and desired physiological conditions
Stability assessment	Applying treatments before community stabilization	Misinterpretation of intervention effects	Verify compositional and metabolic stability before experimental interventions
Microbiota analysis	Relying exclusively on relative abundance data	Incomplete interpretation of microbial changes	Combine sequencing with absolute quantification methods (e.g., qPCR or flow cytometry) and functional analyses
Experimental controls	Absence of baseline or untreated controls	Inability to distinguish spontaneous from treatment-induced changes	Include baseline, negative controls and, where appropriate, positive controls
Reproducibility	Single experiment or single donor only	Limited robustness and generalizability	Include biological and technical replicates and validate findings across independent experiments

Once these methodological foundations have been established, the model can be confidently applied to investigate microbiota-targeted interventions. The following section discusses how validated *in vitro* gut microbiota models can be used to evaluate dietary, probiotic, and pharmacological interventions under controlled experimental conditions.

## 6 Using *in vitro* models to study microbiota modulation

Once an *in vitro* gut microbiota model has been established and validated for stability and reproducibility, it can be used to investigate how external interventions influence microbial community structure and function under controlled conditions. Given the association between gut microbial dysbiosis and metabolic or inflammatory disorders (Zhang Z. et al., 2019; Green et al., 2020), considerable interest has emerged in developing strategies to restore or maintain microbial balance. However, understanding the mechanisms underlying microbiota modulation requires experimental systems capable of isolating microbial responses from complex host-related factors. *In vitro* gut models provide such controlled platforms by enabling the evaluation of specific interventions, including prebiotics (i.e., compounds used as microbial substrates) and probiotics (i.e., live microorganisms) (Green et al., 2020), under standardized and physiologically relevant conditions. These models provide insights into mechanistic links between compounds and microbial transformations, including dose–response relationships and microbiota-dependent functional effects. Collectively, these approaches can support the development of microbiota-informed dietary strategies and personalized probiotic or prebiotic interventions (Abeltino et al., 2024; Shukla et al., 2024). However, *in vitro* models primarily provide mechanistic insights into microbial responses and cannot independently predict clinical outcomes, as host physiology, immune responses, and individual variability remain incompletely represented.

### 6.1 Dietary substrates and prebiotic interventions

Dietary and prebiotic interventions are among the most common applications of biologically relevant *in vitro* gut models because they allow direct investigation of how nutrient availability shapes microbial ecology and metabolic activity. The competitive fermentation of prebiotics by *Bifidobacteria*, alongside other gut microbes, can be effectively studied using *in vitro* gut models (Amaretti et al., 2021). To assess the effects of food components on the HGM, food samples are first subjected to a standardized simulation of upper gastrointestinal digestion using established protocols such as the INFOGEST method (Brodkorb et al., 2019). The non-digested solid residue, along with a defined proportion (typically 10% v/v) of the intestinal soluble fraction, is used as the fermentation substrate, simulating the chyme that reaches the colon (Pérez-Burillo et al., 2021).

Collectively, *in vitro* studies investigating dietary substrates consistently demonstrate that microbial responses are highly dependent on the baseline composition of the gut microbiota (Pérez-Burillo et al., 2021). For example, following *in vitro* fermentation of rye, butyrate production was higher only in microbiota enriched in *Prevotella* and *Subdoligranulum*, whereas Bacteroides-dominated communities showed little increase (Pirkola et al., 2023), highlighting how baseline gut microbiota composition shapes substrate fermentation. This finding confirms that *in vitro* fermentation can effectively capture inter-individual variability in response to nutritional substrates. Maintaining community stability throughout fermentation is therefore essential for correctly interpreting intervention-induced changes.

Other studies using *in vitro* fermentation setups effectively captured the microbial catabolism of poorly absorbed polyphenols such as chlorogenic acid, rutin, and catechins (often found in coffee, tea, and fruits). These experiments have shown their breakdown into phenolic acids like 3,4-dihydroxyphenylacetic acid (Jaganath et al., 2009), ferulic acid (Ludwig et al., 2013), and hydroxyphenylpropionic acid (Roowi et al., 2010; Tomas-Barberan et al., 2014), which exhibit antioxidant and anti-inflammatory properties, further underlining the key role of the microbiota in mediating polyphenol bioactivity. Some other functional substrates, like complex carbohydrates or tea-derived polysaccharides, enhance Bacteroides abundance *in vitro* and increase the production of SCFA by up-regulating carbohydrate-related metabolic pathways (Wang et al., 2019). Notably, high-throughput platforms like the SIFR® reactor demonstrated consistent and reproducible microbiota shifts with prebiotics like inulin, resistant dextrin, and 2′-fucosyllactose; showing concordance with clinical trial outcomes (Van Den Abbeele et al., 2023).

### 6.2 Probiotic interventions

The use of probiotics, including live microbial strains and emerging live biotherapeutic products, in *in vitro* fermentation systems generally involves introducing live microbial strains to evaluate their survival, transient persistence, competitive interactions, and metabolic activity under controlled gut-like conditions (Derrien and Van Hylckama Vlieg, 2015; Marzorati and Van De Wiele, 2016). While these systems are valuable for assessing direct microbe–microbe interactions and functional outputs, they cannot fully reproduce long-term ecological integration, host adhesion processes, immune signaling, or stable colonization dynamics observed *in vivo* (Payne et al., 2012). Consequently, observed probiotic effects should primarily be interpreted as short-term ecological or metabolic responses rather than definitive evidence of sustained colonization or clinical benefit. Interpretation of probiotic effects therefore depends on distinguishing intervention-driven responses from spontaneous microbial changes.

For instance, by using a co-inoculation model, researchers showed that *Bifidobacterium pseudocatenulatum* WC 0401 transiently modulated both the structure and function of the fecal microbiota during fermentation (Quartieri et al., 2016). This highlights community-level probiotic effects, whereas probiotic activity can also occur through specific metabolic transformations. In another model, *Bifidobacterium animalis* was able to hydrolyze chlorogenic acid in the presence of fecal microbiota but without modifying other metabolic pathways, illustrating that probiotic functionality may not always involve broad changes in microbial metabolism (Tomas-Barberan et al., 2014).

In the context of synbiotic interventions, where probiotics are combined with functional prebiotics to enhance microbiota modulation and metabolic output, an *in vitro* batch fermentation study examined the effects of *Bifidobacterium longum* and *Lactobacillus helveticus*, either alone or combined with fructo-oligosaccharides (FOS). Notably, the synbiotic formulation increased butyrate production and modulated profiles of nitrogenous and aromatic volatile compounds. These synergistic effects support the rationale for designing targeted synbiotics aimed at modulating microbial metabolic pathways associated with anti-inflammatory potential (Vitali et al., 2012).

Overall, *in vitro* probiotic studies primarily provide mechanistic insights into microbial survival, competition, and metabolite production, while long-term colonization and host-associated benefits require further validation.

### 6.3 Pharmacological modulators

Fermentation models also provide a powerful platform for investigating how drugs influence gut microbiota structure and function. *In vitro* gut models enable controlled investigation of drug-microbiota interactions by isolating direct microbial responses and removing host-related variables such as absorption, metabolism, and systemic circulation. However, they cannot fully replicate pharmacokinetics, immune signaling, or enterohepatic feedback, and therefore mainly capture direct microbial effects rather than the full bidirectional host-microbiota response. Appropriately validated models are required to distinguish between drug-induced microbial changes and alterations resulting from the culture process itself.

A major observation is that many non-antibiotic drugs exhibit selective antibacterial-like effects. In a study using a high-throughput metaproteomics-based screening platform (called RapidAIM), 35 out of 39 non-antibiotic compounds, including berberine and ibuprofen, were found to modulate the microbial proteome, metabolic function and biomass to varying degrees among different individual microbiotas. Some compounds selectively affected specific microbial taxa without impacting their relative abundance, suggesting that functional modulation can occur independently of compositional shifts (Li et al., 2020). These observations further demonstrate that biologically relevant *in vitro* models should integrate compositional and functional analyses to fully characterize microbiota responses to pharmacological interventions.

Current evidence indicates that pharmacological compounds can either disrupt or restore microbial ecosystem function depending on their mechanism of action. It has been shown that residual antibiotics, even at low doses such as amoxicillin transferred via breast milk, can disrupt early-life microbiota by reducing beneficial taxa (e.g., Lactobacillaceae, Bifidobacteriaceae) and enriching naturally resistant families (Enterococcaceae, Clostridiaceae), while altering metabolic outputs. This highlights long-term risks of indirect antibiotic exposure and the value of *in vitro* models for assessing microbial community vulnerability (Nissen et al., 2022). Another example is lactulose, a non-absorbed disaccharide and common laxative, which was shown in the PathoGut™ SHIME model to suppress *Clostridioides difficile* overgrowth following antibiotic-induced dysbiosis, restore key commensals, boost butyrate production, and normalize bile salt metabolism. This illustrates the potential for certain pharmacological agents to promote microbiota stabilization under controlled *in vitro* conditions, although these effects may not directly translate to *in vivo* settings where host factors and systemic processes also play a major role (Calatayud et al., 2022).

In summary, once microbial communities have been established, *in vitro* fermentation systems provide powerful mechanistic screening platforms for investigating microbiota-targeted interventions. Their main value lies in elucidating how dietary compounds, probiotics, and pharmacological agents influence microbial ecology and metabolic activity under controlled conditions, rather than predicting direct clinical outcomes. Recent advances are expanding their applications from traditional nutrition studies toward pharmacomicrobiomic screening, enabling the investigation of drug-induced microbiota alterations, recovery after antibiotic perturbation, and microbiota-mediated therapeutic effects. However, translation to human health requires integration with host-based models and clinical validation. Importantly, microbiota modulation is often reflected more strongly at the functional and metabolic level than through major taxonomic changes, highlighting the importance of combining compositional profiling with multi-omics approaches (Tian et al., 2020). A comprehensive summary of intervention types, associated microbial responses, and metabolic outcomes is provided in Table 4. Nevertheless, as these applications continue to expand, improving the physiological relevance of *in vitro* models remains essential to ensure that observed microbial responses accurately reflect the complexity of the human gut ecosystem.

**Table 4:** Summary of intervention types evaluated using *in vitro* gut microbiota models and their reported microbial and functional outcomes.

Intervention type	Example compound/substrate	Main microbiota effect	Functional/metabolic outcome
Prebiotic and dietary interventions	Dietary fibers, plant matrices (lentils, quinoa, rye, green tea), inulin, resistant dextrin, 2′-fucosyllactose	Selective shifts in community composition with strong inter-individual variability; enrichment of beneficial taxa depending on baseline microbiota	Increased SCFA production and generation of bioactive metabolites (e.g., phenolic acids); modulation of carbohydrate fermentation pathways; reproducible clinical-aligned microbiota responses (Wang et al., 2019; Amaretti et al., 2021; Pérez-Burillo et al., 2021; Pirkola et al., 2023; Van Den Abbeele et al., 2023)
Polyphenol-rich substrates & bound phenolics	Chlorogenic acid, rutin, catechins; rice bran, grape and carob proanthocyanidins	Microbial biotransformation of polyphenols; increased abundance of beneficial taxa (e.g., Akkermansia, Faecalibacterium)	Production of antioxidant and anti-inflammatory phenolic metabolites (e.g., 3,4-dihydroxyphenylacetic acid, ferulic acid); release of bioactive compounds from fiber-bound forms (Jaganath et al., 2009; Roowi et al., 2010; Saura-Calixto et al., 2010; Ludwig et al., 2013; Tomas-Barberan et al., 2014; Zhang X. et al., 2019)
Probiotic interventions	*Bifidobacterium* and *Lactobacillus* strains	Modulation of microbial community structure and functional activity; strain-specific metabolic interactions	Increased butyrate production and modulation of volatile compound profiles in synbiotic contexts; limited metabolic impact in some single-strain applications; potential effects on mucosal and microbial networks (Vitali et al., 2012; Tomas-Barberan et al., 2014; Quartieri et al., 2016; Raimondi et al., 2021)
Pharmacological modulators	Berberine, ibuprofen, amoxicillin, lactulose	Microbial functional modulation, including protein-expression changes, selective taxonomic shifts, and dysbiosis or recovery depending on the compound	Altered microbial metabolic pathways; suppression or restoration of key commensals; changes in SCFA production and bile salt metabolism; microbiota stabilization or disruption depending on drug type (Li et al., 2020; Calatayud et al., 2022; Nissen et al., 2022)

The methodological considerations discussed throughout the previous sections can be integrated into a practical experimental design framework. Figure 4 summarizes the sequential decisions involved in selecting, configuring, maintaining, validating, and applying biologically relevant *in vitro* human gut microbiota models according to the intended research objective.

**Figure 4:**
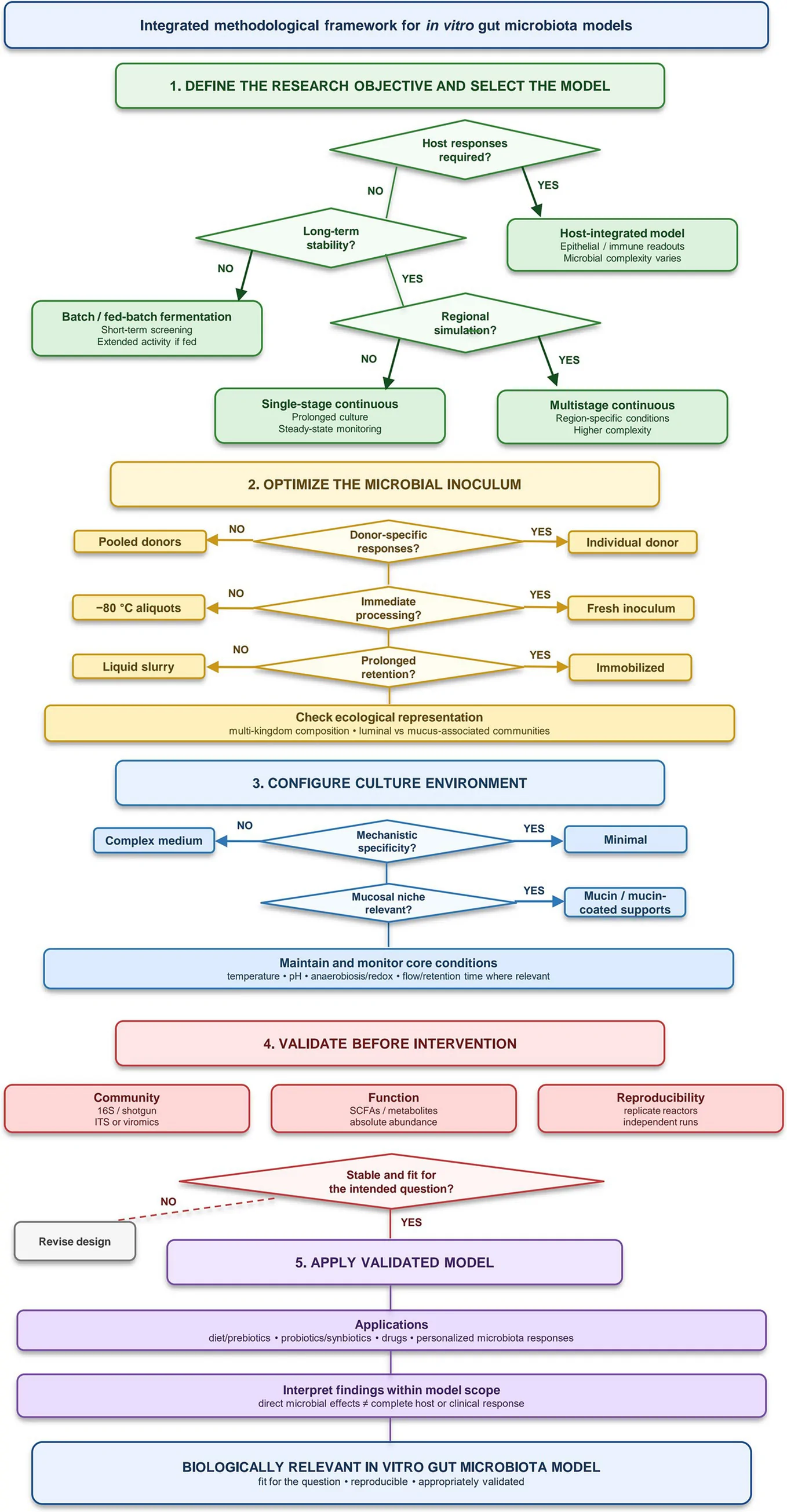
Integrated methodological framework for designing, validating, and applying biologically relevant *in vitro* human gut microbiota models.

## 7 Perspectives and future directions

### 7.1 Current limitations and methodological strengths

Despite considerable advances in the design of *in vitro* gut microbiota models, reproducing the complexity of the human intestinal ecosystem remains a major challenge. Although current systems successfully preserve many aspects of microbial ecology, they cannot yet fully replicate the dynamic interactions between the microbiota, the intestinal epithelium, immune system, and host physiology. As a result, findings derived from simplified *in vitro* systems may not fully predict *in vivo* microbial behavior or host physiological responses, and should therefore be interpreted with caution to avoid overstatement or unwarranted therapeutic extrapolation. Batch experiments are useful for initial screening of treatments on donor communities but fail to represent long-term changes in microbial ecology (Venema and Van Den Abbeele, 2013). Although continuous chemostat systems improve microbial stability and permit long-term experimentation, they still fail to fully replicate the highly dynamic physicochemical gradients of the gastrointestinal tract, including region-specific nutrient fluxes, shear stress, mucus turnover, immune signaling, and oxygen diffusion (Payne et al., 2012). Moreover, the integration of HGM fermentation with host cell models remains underexplored, leaving a critical gap in linking microbial changes to host responses (Isenring et al., 2023). Bridging this gap requires models that increase host physiological representation without unnecessarily sacrificing the ecological complexity of the microbial community.

Another major challenge is the lack of methodological standardization across laboratories. Variability in inoculum preparation, media composition, sequencing pipelines, and analytical workflows complicates cross-study comparisons and limits reproducibility. Addressing this issue will require community-driven guidelines and collaborative benchmarking efforts to harmonize experimental approaches and improve the translational reliability of microbiome research (Sinha et al., 2015).

At the same time, the rise of high-throughput sequencing technologies has transformed our ability to characterize gut microbiota composition and evaluate intervention effects. 16S rRNA gene sequencing enables cost-effective, high-throughput profiling of microbial community structure, allowing monitoring of taxonomic shifts following prebiotic or probiotic treatments (Isenring et al., 2023). Shotgun metagenomics provides deeper species- and strain-level resolution along with the prediction of bacterial functionality, facilitating the link of compositional changes to metabolic pathways and the discovery of previously uncultured bacterial genomes (Payne et al., 2012). Bioinformatic pipelines such as QIIME (Caporaso et al., 2010), MetaPhlAn (Segata et al., 2012), and Kraken2 (Lu et al., 2026) facilitate the processing and interpretation of microbiome sequencing data by transforming raw sequencing reads into taxonomic profiles, community diversity metrics, and, when combined with functional profiling approaches, predictions of microbial metabolic potential. When integrated with complementary *in vitro* models, multi-omics approaches provide a comprehensive framework for dissecting microbial dynamics and functional responses, offering the possibility to investigate mechanisms and translational aspects. These technologies therefore complement biologically relevant fermentation models by providing compositional and functional information that cannot be inferred from microbial growth or metabolite measurements alone.

### 7.2 Integrating host physiology into fermentation models

Although current fermentation models can reproduce important aspects of microbial ecology under controlled conditions, their main limitation remains the absence of host-derived feedback mechanisms. However, microbial ecological complexity and host physiological relevance should be considered as distinct dimensions of model performance. Conventional fecal fermentation systems may maintain highly diverse microbial communities and complex microbe–microbe interactions while reproducing relatively few host processes. Conversely, host-integrated platforms such as organoids and gut-on-chip systems can reproduce epithelial architecture, mechanical forces, oxygen gradients, and host signaling more effectively, but often accommodate less complex microbial communities or defined microbial consortia. Increasing host complexity therefore does not necessarily increase microbial ecological fidelity, and the appropriate balance between these dimensions depends on the biological question being addressed. Integrating epithelial, immune, and metabolic responses therefore represents an important strategy for extending the range of host-related processes that can be investigated *in vitro*. The combination of gut fermentation systems with human intestinal cell cultures enables simultaneous investigation of microbial dynamics and host responses, providing a more comprehensive representation of host–microbiota interactions.

With the development of gut-on-chip technologies based on microfluidics, several host-associated features of the intestinal environment can be reproduced more precisely *in vitro.* By integrating oxygen gradients, microbial-epithelial co-culture and constant flow together with mechanical stimulation that can approximate aspects of intestinal peristalsis within microengineered chambers, these platforms provide greater host physiological complexity than conventional static microbial cultures (Jalili-Firoozinezhad et al., 2019; Xiang et al., 2020). Gut-on-chip platforms have already been successfully employed for evaluating host inflammatory signaling and microbe-induced changes in intestinal barrier permeability under flow, highlighting their ability to reproduce host-associated processes that are absent from conventional fermentation systems (Kim et al., 2012; Shin and Kim, 2018). Integrating these models with multi-omics readouts provides a significant step to overcome the existing gap between conventional fermentation systems and more complex tissue-based models. Nevertheless, gut-on-chip platforms remain technically demanding, expensive, and difficult to standardize across laboratories. Long-term maintenance of complex anaerobic microbial communities alongside oxygen-dependent epithelial tissues remains particularly challenging. As a result, many host-integrated systems still rely on individual strains or relatively simplified microbial consortia rather than donor-derived communities approaching the ecological complexity supported by dedicated fermentation platforms. Furthermore, integration of immune, neuronal, vascular, and endocrine components into a single reproducible platform is still in early development (Bein et al., 2018).

Beyond host-integrated platforms, next-generation fermentation systems are also being developed to improve ecological control and experimental reproducibility. These approaches combine advanced cultivation strategies, synthetic microbial communities, and computational modeling to better define how specific microbial components influence ecosystem stability and function. When integrated with multi-omics and systems biology approaches, these models may contribute to more predictive frameworks for microbiota-targeted interventions (Payne et al., 2012).

### 7.3 Next-generation host–microbe co-culture models

Organoid-based systems represent a complementary strategy for increasing host physiological representation in studies of direct host-microbiota interactions. Organoids are self-organizing, three-dimensional structures derived from stem cells which mimic the structural and physiological characteristics of the gut, such as epithelial polarity and the formation of a low-oxygen luminal space (Biagini et al., 2023). Early co-culture strategies involved microinjection of microbial communities into the organoid lumen, enabling controlled analysis of host–microbe interactions within a physiologically relevant epithelial system (Walocha et al., 2024).

These systems provide important opportunities to study how microbial composition influences host physiology and how dietary compounds, drugs, or pathogens modulate microbial activity. However, several challenges remain, such as the maintenance of stable and complex microbial communities, the reproduction of physiological oxygen gradients, and the control of microbial metabolite toxicity. Furthermore, existing models are predominantly focused on defined consortia or single strains rather than complex gut ecosystem diversity. Their increased representation of epithelial physiology should therefore not be interpreted as equivalent to increased microbial ecological complexity. Instead, organoid-based and fermentation models provide complementary information, with the former prioritizing host cellular responses and the latter generally supporting more complex community-level microbial ecology. Future advancements should be focused on improving long-term microbial stability and integrating predictive in silico models to enhance system interpretability (Puschhof et al., 2021).

Recent innovations further expand this field. Dynamic luminal culture systems combined with epithelial co-culture models allow more realistic simulation of gut–microbe interactions under controlled conditions (Venema and Van Den Abbeele, 2013). Intestinal stem cells can now be differentiated into crypt-like organoids containing multiple specialized cell types, including enterocytes, goblet cells, and Paneth cells, closely mimicking *in vivo* intestinal architecture (Almeqdadi et al., 2019). Additionally, advanced 3D culture platforms, such as rotating wall vessel bioreactors, enable the formation of more complex epithelial structures that support prolonged microbial exposure and interaction (Salerno-Goncalves et al., 2016). Together, these approaches expand the range of biological questions that can be addressed *in vitro* by complementing microbial-community models with increasingly sophisticated representations of host physiology. Rather than replacing conventional fermentation systems, host-integrated platforms provide an additional layer of biological information, particularly when epithelial responses, barrier function, or host signaling are central to the experimental objective. Future model development should therefore focus on combining host physiological representation with the ecological complexity and reproducibility achieved in dedicated microbial fermentation systems.

### 7.4 Toward precision microbiome modeling

The ultimate objective of developing biologically relevant *in vitro* gut microbiota models is to move toward precision microbiome research, where individual microbial characteristics can be considered when evaluating responses to dietary, probiotic, and pharmacological interventions. Future advances may enable the development of personalized *in vitro* systems capable of predicting microbiota-specific responses to external perturbations and identifying optimal strategies for microbiota modulation. Achieving this goal will require the integration of biologically representative models with multi-omics approaches, advanced computational methods, and artificial intelligence-driven analyses to transform complex microbiome data into predictive frameworks (Singh and Singh, 2026). Ultimately, these developments will help bridge the gap between mechanistic microbiome research and personalized interventions for human health.

Future developments will likely combine biologically representative fermentation systems with artificial intelligence, machine learning, and computational modeling approaches (Tourab et al., 2026). These tools may enable the integration of microbiota composition, metabolic outputs, and host-derived parameters to generate predictive models of individual responses to dietary or pharmacological interventions. Digital twin concepts, incorporating longitudinal microbiome and clinical data, could eventually support personalized simulation of microbiota dynamics and optimize intervention strategies before clinical application (Vallée, 2026). Additional advances in spatial microbiology, real-time metabolomics, and synthetic microbial communities will further improve the ability to monitor and manipulate complex microbial ecosystems. However, achieving truly predictive microbiome models will require not only technological innovation but also standardized reference communities, harmonized experimental workflows, and validation against human datasets.

## 8 Conclusion

*In vitro* models of the human gut microbiota are indispensable tools for investigating microbial ecology, metabolism, and responses to external interventions under controlled conditions. This review highlights that their biological relevance depends on the integration of multiple methodological decisions rather than on the choice of fermentation platform alone. Appropriate model selection, inoculum preparation, culture medium composition, environmental control, and rigorous validation of microbial stability and reproducibility collectively determine the reliability and interpretability of experimental outcomes.

By synthesizing current evidence into an integrated methodological framework, this review provides practical guidance for the design, validation, and application of biologically relevant *in vitro* gut microbiota models. Such an approach may contribute to improving experimental reproducibility, facilitating comparisons between studies, and supporting the development of standardized methodologies across laboratories.

Emerging technologies, including host-integrated culture systems, multi-omics approaches, and computational modeling, are expected to further improve the physiological relevance and predictive capacity of these models. Nevertheless, *in vitro* systems should continue to be regarded as complementary mechanistic platforms that support, rather than replace, animal and human studies.

Ultimately, future progress will depend not only on technological innovation but also on the establishment of consensus methodologies and reporting standards that enhance reproducibility and comparability across laboratories. By combining methodological rigor with increasing biological complexity, *in vitro* gut microbiota models will continue to evolve into robust experimental platforms that advance mechanistic microbiome research and accelerate the development of precision microbiome interventions.
